# Increased risk of kidney failure in patients with genetic kidney disorders

**DOI:** 10.1172/JCI178573

**Published:** 2024-09-03

**Authors:** Mark D. Elliott, Natalie Vena, Maddalena Marasa, Enrico Cocchi, Shiraz Bheda, Kelsie Bogyo, Ning Shang, Francesca Zanoni, Miguel Verbitsky, Chen Wang, Victoria Kolupaeva, Gina Jin, Maayan Sofer, Rafael Gras Pena, Pietro A. Canetta, Andrew S. Bomback, Lisa M. Guay-Woodford, Jean Hou, Brenda W. Gillespie, Bruce M. Robinson, Jon B. Klein, Michelle N. Rheault, William E. Smoyer, Larry A. Greenbaum, Larry B. Holzman, Ronald J. Falk, Afshin Parsa, Simone Sanna-Cherchi, Laura H. Mariani, Matthias Kretzler, Krzysztof Kiryluk, Ali G. Gharavi, CureGN Consortium

**Affiliations:** 1 Department of Medicine, Division of Nephrology, Vagelos College of Physicians & Surgeons, Columbia University, New York, New York, USA.; 2 University of British Columbia, Vancouver, British Columbia, Canada.; 3 Neonatal and Pediatric Intensive Care Unit, Bufalini Hospital, AUSL Romagna, Ravenna, Italy.; 4 University of Pennsylvania, Philadelphia, Pennsylvania, USA.; 5 Department of Pediatrics, Division of Nephrology, Children’s Hospital of Philadelphia, Philadelphia, Pennsylvania, USA.; 6 Department of Laboratory Medicine and Pathology, Cedars Sinai Medical Center, Los Angeles, California, USA.; 7 Department of Biostatistics, School of Public Health, and; 8 Department of Internal Medicine, Division of Nephrology, University of Michigan, Ann Arbor, Michigan, USA.; 9 Department of Internal Medicine, Division of Nephrology and Hypertension, University of Louisville School of Medicine, Louisville, Kentucky, USA.; 10 Robley Rex VA Medical Center, Louisville, Kentucky, USA.; 11 Department of Pediatrics, University of Minnesota, Minneapolis, Minnesota, USA.; 12 Department of Pediatrics, The Research Institute at Nationwide Children’s Hospital, The Ohio State University, Columbus, Ohio, USA.; 13 Department of Pediatrics, Division of Pediatric Nephrology, Emory University School of Medicine and Children’s Healthcare of Atlanta, Atlanta, Georgia, USA.; 14 Perelman School of Medicine, Division of Nephrology, University of Pennsylvania, Philadelphia, Pennsylvania, USA.; 15 Department of Medicine, Division of Nephrology, University of North Carolina, Chapel Hill, North Carolina, USA.; 16 Division of Kidney, Urologic & Hematologic Diseases, National Institute of Diabetes and Digestive and Kidney Diseases, NIH, Bethesda, Maryland, USA.; 17 Institute for Genomic Medicine, Columbia University, New York, New York, USA.; 18 The CureGN Consortium is detailed in Supplemental Acknowledgments.

**Keywords:** Genetics, Nephrology, Chronic kidney disease, Genetic diseases, Molecular diagnosis

## Abstract

**BACKGROUND:**

It is unknown whether the risk of kidney disease progression and failure differs between patients with and without genetic kidney disorders.

**METHODS:**

Three cohorts were evaluated: the prospective Cure Glomerulonephropathy Network (CureGN) and 2 retrospective cohorts from Columbia University, including 5,727 adults and children with kidney disease from any etiology who underwent whole-genome or exome sequencing. The effects of monogenic kidney disorders and *APOL1* kidney-risk genotypes on the risk of kidney failure, estimated glomerular filtration rate (eGFR) decline, and disease remission rates were evaluated along with diagnostic yields and the impact of American College of Medical Genetics secondary findings (ACMG SFs).

**RESULTS:**

Monogenic kidney disorders were identified in 371 patients (6.5%), high-risk *APOL1* genotypes in 318 (5.5%), and ACMG SFs in 100 (5.2%). Family history of kidney disease was the strongest predictor of monogenic disorders. After adjustment for traditional risk factors, monogenic kidney disorders were associated with an increased risk of kidney failure (hazard ratio [HR] = 1.72), higher rate of eGFR decline (–3.06 vs. 0.25 mL/min/1.73 m^2^/year), and lower risk of complete remission (odds ratio_Not_ _achieving_ _CR_ = 5.25). High-risk *APOL1* genotypes were associated with an increased risk of kidney failure (HR = 1.67) and faster eGFR decline (–2.28 vs. 0.25 mL/min/1.73 m^2^), replicating prior findings. ACMG SFs were not associated with personal or family history of associated diseases, but were predicted to impact care in 70% of cases.

**CONCLUSIONS:**

Monogenic kidney disorders were associated with an increased risk of kidney failure, faster eGFR decline, and lower rates of complete remission, suggesting opportunities for early identification and intervention based on molecular diagnosis.

**TRIAL REGISTRATION:**

NA.

**FUNDING:**

National Institute of Diabetes and Digestive and Kidney Diseases grants U24DK100845 (formerly UM1DK100845), U01DK100846 (formerly UM1DK100846), U01DK100876 (formerly UM1DK100876), U01DK100866 (formerly UM1DK100866), U01DK100867 (formerly UM1DK100867), U24DK100845, DK081943, RC2DK116690, 2U01DK100876, 1R01DK136765, 5R01DK082753, and RC2-DK122397; NephCure Kidney International; Department of Defense Research Awards PR201425, W81XWH-16-1-0451, and W81XWH-22-1-0966; National Center for Advancing Translational Sciences grant UL1TR001873; National Library of Medicine grant R01LM013061; National Human Genome Research Institute grant 2U01HG008680.

## Abstract 2

Across 3 cohorts, monogenic kidney disorders were associated with an increased risk of kidney failure, higher eGFR decline rate, and lower rates of complete remission.

## Graphical abstract

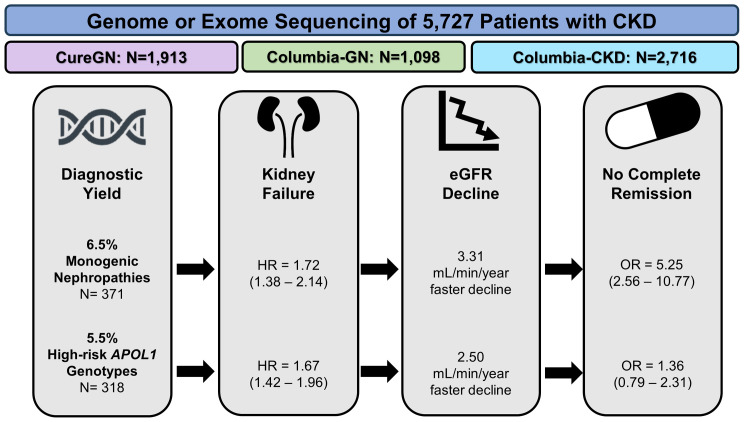

## Introduction

Chronic kidney disease (CKD) is a heterogeneous group of conditions affecting over 10% of the population, causing substantial morbidity and mortality (1–3). Genetic kidney disorders are well-recognized causes of pediatric-onset kidney disease, and are increasingly recognized as important causes of adult-onset CKD (4, 5). Patients with a family history of kidney disease, early onset, extra-renal symptoms, or specific phenotypes have higher diagnostic rates of genetic kidney disorders (5, 6).

Diagnosing genetic kidney disorders is crucial for clinical management, family planning, transplant decision making, and providing patients with a specific disease etiology (6–9). For example, remission is felt to be unlikely for most monogenic glomerular diseases, but a few disorders may respond to therapy (10–12). Therapies targeted to specific genetic disorders are being developed, increasing the importance of making a genetic diagnosis (13–16). Patients are increasingly advocating for improved access to genetic testing, and find the results useful when making health decisions (17–19).

Much of what we know about genetic kidney disorders comes from small case series, and limited data exist evaluating the clinical outcomes of patients with genetic kidney disorders in larger cohorts (20–23). Therefore, we performed diagnostic genetic analysis based on whole-genome or exome sequence data in 3 cohorts, and evaluated the clinical outcomes of individuals with monogenic kidney disorders and *APOL1* kidney risk genotypes. We also examined predictors of monogenic kidney disorders, and the impact of American College of Medical Genetics and Genomics secondary findings (ACMG SFs) on personal and family history of associated conditions and kidney disease management.

## Results

### Diagnostic yield.

In the Cure Glomerulonephropathy Network (CureGN), 53 participants (2.8%) had a monogenic glomerular disorder, defined by a diagnostic finding in glomerular disease–related genes in Supplemental Table 1 (supplemental material available online with this article; https://doi.org/10.1172/JCI178573DS1); the majority had a biopsy diagnosis of focal segmental glomerulosclerosis (FSGS) (33 participants). Additionally, 122 participants (6.4%) had high-risk *APOL1* genotypes, and 100 (5.2%) had ACMG SFs (Tables 1 and 2 and Supplemental Tables 2, 3, and 4). In the Columbia-GN cohort, we identified 39 (3.6%) individuals with a monogenic glomerular disorder, mainly in patients with FSGS (35 individuals), along with 66 individuals (6.0%) with high-risk *APOL1* genotypes. In the Columbia-CKD cohort, 279 individuals (10.3%) had a monogenic kidney disorder, defined by a diagnostic finding in any kidney disease gene in Supplemental Table 1, and 130 (4.8%) had high-risk *APOL1* genotypes (Table 2 and Supplemental Table 2). Few participants had both a monogenic kidney disorder and a high-risk *APOL1* genotype, with the majority being in type 4 collagen genes (5 CureGN participants, 1 Columbia-GN participant, and 6 Columbia-CKD participants; Supplemental Table 2).

Family history of kidney disease was most consistently associated with monogenic kidney disorders (odds ration [OR] = 2.80, 1.69, and 3.59; *P* = 4.61 × 10^–4^, 0.14, and *P* < 2 × 10^–16^ in CureGN, Columbia-GN, and Columbia-CKD, respectively; Supplemental Table 5). In CureGN, monogenic glomerular disorders were most common in individuals with FSGS, and less frequent in those with membranous nephropathy (MN) or who self-reported as Black/African American (OR = 2.78, 0.18, and 0.28; *P* = 5.62 × 10^–3^, 0.029, and 0.019, respectively). In the Columbia-CKD cohort, monogenic kidney disorders were identified more frequently in individuals with diagnoses of congenital or Mendelian kidney disease, tubulointerstitial disease, glomerular disorders not included in CureGN, or kidney disease of unknown etiology when compared with those with diabetic kidney disease (OR = 6.97, 3.91, 3.81, and 3.64; *P* = 2.43 × 10^–7^, 7.77 × 10^–3^, 4.89 × 10^–4^, and 1.17 × 10^–3^, respectively). High-risk *APOL1* genotypes were more common in individuals with FSGS (OR = 8.86 and 5.27; *P* = 4.19 × 10^–9^ and 0.033 in CureGN and Columbia-GN, respectively), hypertension-associated kidney disease (OR = 2.12, *P* = 0.024), and those who self-identified as Black/African American (OR = 167, 28.88, and 16.92; *P* = 3.49 × 10^–25^, <2 × 10^–16^, and <2 × 10^–16^ in CureGN, Columbia-GN, and Columbia-CKD, respectively) or Latinx (OR = 2.46, 6.03, and 1.83; *P* = 0.047, 1.70 × 10^–5^, and 0.014 in CureGN, Columbia-GN, and Columbia-CKD, respectively; Supplemental Table 5). No associations were identified between *APOL1* risk genotype and monogenic kidney disorders. The proportion of patients on immunosuppression was lower in patients with monogenic kidney disorders in all 3 cohorts, as was renin-angiotensin blockade in both Columbia-GN and Columbia-CKD.

### Clinical outcomes.

**Figure 1:**
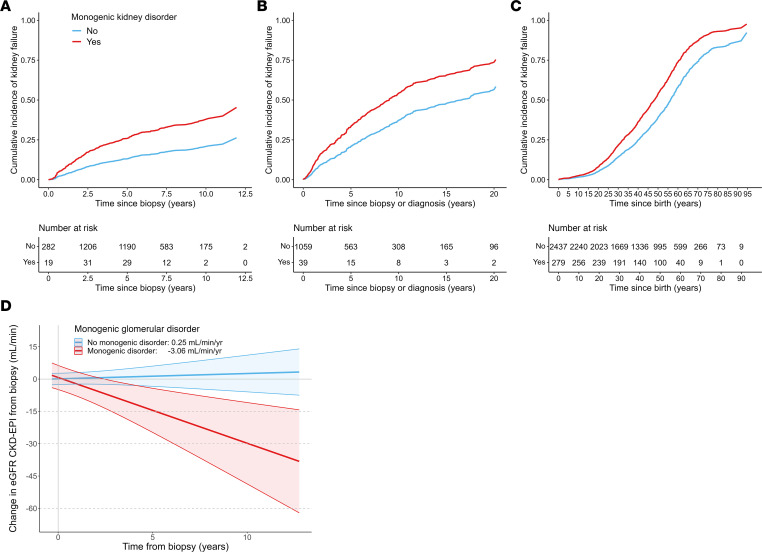
Conditionally adjusted event curves showing kidney failure rates based on monogenic kidney disorders in the CureGN cohort (**A**) measured from kidney biopsy, Columbia-GN cohort (**B**) measured from kidney biopsy or clinical diagnosis, and Columbia-CKD cohort (**C**) including risk tables. Rate of eGFR decline in CureGN based on the presence of monogenic glomerular disorder from time of kidney biopsy reported as mean and 95% confidence interval (**D**). CKD-EPI, Chronic Kidney Disease Epidemiology Collaboration.

**Figure 2:**
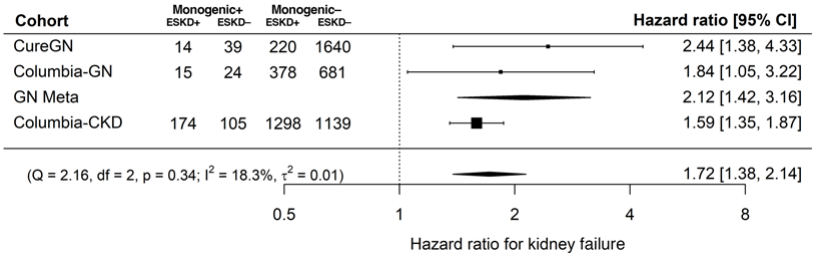
Restricted maximum likelihood random-effects meta-analysis of kidney failure risk across all 3 cohorts evaluating the effect of monogenic glomerular disorders using the fully adjusted Cox models. Subanalysis of genetic glomerular disorders within CureGN and Columbia-GN is also included. ESKD, end-stage kidney disease.

In all 3 cohorts, individuals with monogenic kidney disorders experienced an increased risk of kidney failure compared with those without (CureGN: hazard ratio [HR] = 2.44, *P* = 2.42 × 10^–3^; Columbia-GN: HR = 1.84, *P* = 0.033; Columbia-CKD: HR = 1.59, *P* = 2.06 × 10^–8^; Table 3, Figure 1, and Supplemental Figure 1). Meta-analysis showed minimal heterogeneity (HR = 1.72, *P* = 1.47 × 10^–6^, *Q* = 2.16, *P*_Heterogeneity_ = 0.341, *I*^2^ = 18.3%, τ^2^ = 0.01; Figure 2), as did the subgroup analysis of the CureGN and Columbia-GN cohorts (HR = 2.12, *P* = 2.86 × 10^–4^, *Q* = 0.42, *P*_Heterogeneity_ = 0.52, *I*^2^ = 0.0%, τ^2^ = 0.00; Figure 2). A sensitivity analysis of the Columbia cohort with the inclusion of urine albumin to creatinine ratio (UACR) and estimated glomerular filtration rate (eGFR) as covariates showed a consistent magnitude and direction of effect, despite a reduced sample size (Supplemental Table 6 and Supplemental Figure 2).There was a consistent direction of effect for monogenic kidney disorders on the risk of kidney failure across the specific glomerular subgroups; however, only CureGN IgA nephropathy/IgA vasculitis (IgAN) and Columbia-GN FSGS reached statistical significance (HR = 4.84 and 1.89; *P* = 0.022 and 0.043, respectively; Supplemental Table 7).

In CureGN, individuals with monogenic glomerular disorders had higher rates of eGFR decline than those without (–3.06 vs. 0.25 mL/min/1.73 m^2^/year, *P* = 2.63 × 10^–4^; Figure 1) and were less likely to achieve complete remission (CR) (OR_Not_ _achieving_ _CR_ = 5.25, *P* = 6.31 × 10^–6^; Supplemental Table 8). Complete case sensitivity analysis within the CureGN cohort showed consistent effect size and direction for kidney failure and CR risk, and eGFR decline rate, and both analyses confirmed known risk factors of progression, including baseline eGFR, urine protein to creatinine ratio (UPCR), and carrying a diagnosis of hypertension or FSGS (Supplemental Table 9) (24, 25).

In Columbia-GN and Columbia-CKD, high-risk *APOL1* genotypes were associated with an increased risk of kidney failure, while CureGN showed a nonsignificant trend toward increased risk (HR = 1.72, 1.74, and 1.28; *P* = 0.018, 1.14 × 10^–6^, and 0.31, respectively; Table 3). Limiting the analysis of the effect of high-risk *APOL1* genotypes in CureGN to only those genetic ancestry clusters with individuals with high-risk *APOL1* continued to show a nonsignificant trend toward increased risk (HR = 1.49, *P* = 0.14). Strong correlations between *APOL1* kidney risk genotype and genetic ancestry cluster reduced the size and strength of this association (Supplemental Figure 3). There was no effect of the interaction between *APOL1* kidney risk genotype and monogenic kidney disorders on the likelihood of kidney failure (*P* = 0.45). Meta-analysis of the effect of specific high-risk *APOL1* genotypes showed an increased risk of kidney failure without heterogeneity between the 3 high-risk *APOL1* genotypes, or across the 3 studies (HR = 1.67, *P* = 9.13 × 10^–10^; Supplemental Figure 4). In CureGN, individuals with high-risk *APOL1* genotypes experienced a higher rate of eGFR decline (–2.28 vs. 0.25 mL/min/1.73 m^2^/year, *P* = 4.35 × 10^–4^; Supplemental Figure 5), but a similar rate of CR when compared to individuals with low-risk *APOL1* genotypes (OR_Not_ _achieving_ _CR_ = 1.36, *P* = 0.27; Supplemental Table 8).

### ACMG SFs.

Within the 100 CureGN individuals with ACMG SFs, we did not identify an enrichment of personal or family histories of clinical features associated with respective SFs (Supplemental Table 10). As expected, there was no association between ACMG SF and kidney failure (HR = 0.88, *P* = 0.70). However, for 70 of the ACMG SF carriers, there was a predicted impact on the kidney care they would receive, such as the management of immunosuppression in patients with genetic predisposition to cancer (Supplemental Table 3).

## Discussion

Genetic analysis of 5,727 patients with kidney disease from 3 diverse cohorts identified 371 patients (6.5%) with monogenic kidney disorders, 318 (5.5%) with high-risk *APOL1* genotypes, and 100 (5.2%) in CureGN with ACMG SFs. Family history was a predictor of monogenic kidney disorders, confirming prior studies (5, 6, 20). The highest diagnostic yield for monogenic glomerular disorders (8.7%) and high-risk *APOL1* genotypes (19%) was observed in patients with FSGS, as expected given the rarity of monogenic forms of non-FSGS glomerulopathies. This likely explains the lower diagnostic yield compared with other studies of patients with kidney disease (6, 8, 20, 26). Reassuringly, our rate of ACMG SF diagnoses is in line with other studied populations (27–29).

Recent reports have detected reduced life span in individuals with ACMG SF genotypes and carriers of large structural genomic variants (29, 30). Here, we show that individuals with monogenic kidney disorders have an increased risk of kidney failure, a higher rate of eGFR decline, and are less likely to achieve CR, independent of traditional risk factors (31–35). Within the CureGN glomerular diagnoses, we found a consistent direction and size of effect of monogenic disorders on kidney failure risk. Similarly, high-risk *APOL1* genotypes were associated with an increased risk of kidney failure and higher eGFR decline rate, but no difference in CR rates, consistent with prior studies (36, 22, 37, 21). No interaction was identified between monogenic kidney disorders and *APOL1* kidney risk genotype, but our analysis is likely underpowered in this assessment. Our analysis confirmed the effect of traditional risk factors, including hypertension, on the risk of kidney failure, consistent with prior cohort studies (24, 25).

These data suggest that recognition of genetic kidney disorders will be important for clinical evaluation, understanding prognosis, and counseling for patients. While targeted therapies are available only for a minority of monogenic kidney diseases, a genetic diagnosis may still impact management by supporting cessation of inefficacious therapies such as immunosuppression for monogenic podococytopathies and the identification of unrecognized extrarenal manifestations (7). The data suggest that inclusion of patients with unrecognized genetic kidney disorders may skew clinical trial outcomes toward negative results. Therefore, implementing genetically stratified trials may improve the assessment of interventions on outcomes like progression and remission. Finally, systematic identification of monogenic kidney disorders will provide a more accurate assessment of prevalence and feasibility of clinical trials for specific genetic disorders, and thus may encourage research into novel targeted therapies.

There are several potential explanations for worse outcomes in patients with monogenic kidney diseases. Genetic diseases involve exposure to structural, physiologic, or metabolic defects from birth, which increase the duration of disease burden. Moreover, many genetic glomerular disorders involve structural defects of the podocytes or the glomerular basement membranes that are typically unresponsive to immunosuppression, unlike autoimmune glomerular disorders. There is evidence of benefit of nonspecific therapies, like renin-angiotensin system inhibitors, in genetic kidney diseases; however, these may be less beneficial for patients with genetic kidney diseases compared with those without (38–40). These possibilities will have to be studied in larger cohorts, but suggest that early recognition of monogenic kidney diseases and initiation of therapies before pronounced eGFR decline may reduce progression to end-stage kidney disease.

No increased risk was found for phenotypes associated with ACMG SFs, nor of kidney failure in those with ACMG SFs, in CureGN. However, the analysis was limited by the small number of individuals with each ACMG SF, limited follow-up time, and limited data for non-kidney outcomes. Given these are actionable genetic findings, it is expected that all 100 ACMG SFs will lead to changes in some aspect of clinical care. Longitudinal follow-up of participants will enable better assessment of outcomes associated with ACMG SFs. Additionally, patients who developed SF-associated phenotypes, like malignancy or early-onset diabetes, may have been excluded from study enrollment. Nevertheless, the majority of these findings were predicted to impact management of the underlying kidney disease; however, the actual impacts on care were not directly assessed. These highlight the potential for developing a list of actionable genetic findings for kidney care beyond monogenic kidney diagnoses.

We did not survey intronic and intergenic variants, copy number variations, and variants in difficult-to-sequence regions of genes such as the known tandem repeat domain in *MUC1* (6, 41). This may result in an underestimation of the diagnostic yield of these disorders and potentially underestimate the risk of kidney failure and progression, due to undiagnosed cases being classified as a non-monogenic kidney disorders. Other limitations include the enrollment into CureGN after biopsy and the retrospective nature of the Columbia biobank, introducing potential confounders or biases. Despite the relatively large cohorts analyzed, few individuals share specific pathogenic variants, or even affected genes. This limits our ability to assess granular outcome data at the gene level, potentially leading to a less precise grouping of different conditions. The high missingness of eGFR and UPCR measurements in the Columbia cohorts meant these known prognostic markers were excluded from the primary analysis. Given their clinical importance, we performed a sensitivity analysis using the available data, which showed consistent risk effect estimates despite smaller cohort size (Supplemental Table 6, Supplemental Figure 2). The wide range of kidney diseases included in the cohorts and the multicenter nature of CureGN allow these results to be generalizable, but the rarity of specific diagnoses limits our ability to evaluate specific genetic kidney disorders in these cohorts. Moreover, our cohorts may not be completely representative of CKD populations, as individuals with diabetes mellitus and hypertension-associated nephropathy are relatively underrepresented.

This study confirmed the meaningful diagnostic yield of genetic testing for patients with kidney disease and showed that monogenic kidney disorders are associated with an increased risk of kidney failure, higher rate of eGFR decline, and lower rates of CR. Furthermore, it confirmed the association between high-risk *APOL1* genotypes and kidney failure and eGFR decline. These support the importance of genetic testing for risk stratification and counseling for patients with kidney disease and motivate earlier intervention and a search for more effective therapies for genetic kidney disorders.

## Methods

### Sex as a biologic variable.

Participants of this study were of male and female sex. Sex was used as a covariate in the adjusted analyses, as women are more commonly affected by CKD; however, men experience an increased risk of disease progression and kidney failure (42).

### Study design and cohorts.

**Figure 3:**
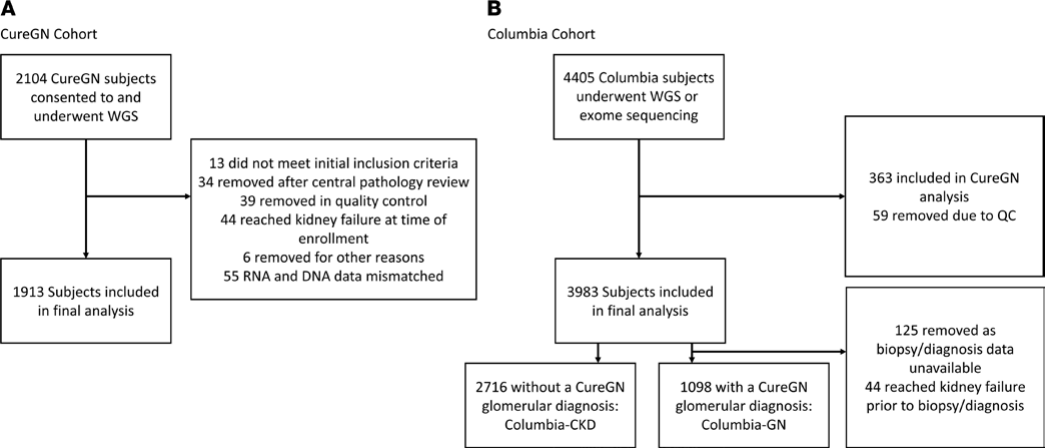
Cohort outline and flow diagram. (**A**) CureGN cohort. WGS, whole-genome sequencing. (**B**) Columbia cohort. Cohort definitions. CureGN: This is the cohort of individuals from the CureGN study who have biopsy-proven minimal change disease (MCD), focal segmental glomerulosclerosis (FSGS), IgA nephropathy or IgA vasculitis (IgAN), or membranous nephropathy (MN). Columbia-GN: This is a cohort of individuals from the Columbia University genetic studies of the CKD biobank who have a diagnosis of 1 of the 4 glomerular disorders included in CureGN. These include MCD, FSGS, IgAN, and MN. Columbia-CKD: This is the remaining individuals from the Columbia University genetic studies of the CKD biobank who have any diagnosis other than the 4 glomerular disorders in CureGN. This includes structural and cystic kidney disease, tubulointerstitial diseases, and other glomerular disorders that are not MCD, FSGS, IgAN, or MN.

CureGN recruited children and adults within 5 years of a kidney biopsy who showed minimal change disease (MCD), FSGS, MN, or IgAN from 71 sites across the United States, Canada, Poland, and Italy, beginning in December 2014 (43). Participants with chronic dialysis, kidney transplant, diabetes mellitus, lupus erythematosus, human immunodeficiency virus, active malignancy, or hepatitis B or C at time of first kidney biopsy were excluded. Among 2,104 CureGN participants who consented for genome sequencing, 1,913 were included after quality control (QC; Figure 3). Data were collected prospectively from enrollment and retrospectively to the time of kidney biopsy. The mean length of follow-up was 4.94 years. Missing clinical data were imputed using chained equations with the mice package in R (44). Race and ethnicity were self-reported or reported by parents of children as mandated by the US NIH, consistent with the Inclusion of Women, Minorities, and Children policy. Missing and unknown race and ethnicity data was due to a mix of participant nonresponse and preference not specified. Definitions for the 3 cohorts are provided in the Figure 3 legend.

The genetic studies of the CKD biobank included 4,405 Individuals with CKD recruited at New York Presbyterian Hospital/Columbia University Irving Medical Center (CUIMC) in New York City, USA, between October 2013 and October 2022. Participants underwent exome or genome sequencing following standard protocols at the Institute for Genomic Medicine at Columbia University (6, 45). After QC and excluding individuals included in CureGN, 3,914 individuals remained, including 1,098 individuals with a glomerular diagnosis included in CureGN (Columbia-GN cohort) and 2,716 individuals with a different clinical diagnosis (Columbia-CKD cohort; Figure 3). Mean follow-up times were 2.65 and 1.99 years, respectively. Retrospective clinical data were collected from the electronic health records. Due to use of the same biobank data set, 142 individuals in CureGN, 619 in Columbia-GN, and 1,294 in Columbia-CKD were included in Groopman et al. (6).

### Genetic analysis.

Genome sequencing was performed using the Illumina NovaSeq 6000 platform. Sequencing data were analyzed using Analysis Tool for Annotated Variants (ATAV) to identify variants diagnostic of an individual’s kidney disease using a curated list of 115 genes known to cause monogenic glomerular disorders, a broader list of 753 genes known to cause nonglomerular monogenic kidney disease, and ACMG SFs using the V3.1 list (Supplemental Table 1) (46–49). Variant interpretation followed the ACMG and Association for Molecular Pathology guidelines with updates from the Clinical Genomics Resource sequence variant interpretation working group, and disease-specific recommendations (50–53). Diagnostic variants were reviewed at multidisciplinary genetic sign-out rounds and reached consensus interpretation. They are considered returnable after Clinical Laboratory Improvement Amendments–certified lab confirmation. *APOL1* risk genotypes were defined by the presence of 2 of the G1 (G1_G_ and G1_M_) or G2 kidney risk alleles. Genetic ancestry clustering was performed using Leiden clustering of 12,400 informative genetic ancestry markers (54).

### Statistics.

A *P* value of less than 0.05 was considered significant. The primary analysis examined the effect of monogenic kidney disorders on the risk of kidney failure, defined as the initiation of chronic dialysis or kidney transplantation, using Cox’s proportional hazard models within each cohort and meta-analyzed as below (55). The proportional hazards assumption was met for each model.

Four models were evaluated in CureGN using the imputed data set, with the start time being the kidney biopsy (a) unadjusted model; (b) minimally adjusted model that included sex, pathologic diagnosis, age at biopsy, and *APOL1* kidney risk genotype; (c) matching adjusted model that added hypertension, diabetes, and use of immunosuppression at time of biopsy, use of renin angiotensin aldosterone system (RAAS) inhibitor at enrollment, and genetic ancestry cluster to the minimally adjusted model so the covariates included matched those in CUIMC as closely as possible; and (d) fully adjusted that added eGFR and UPCR (in g/g) at the time of biopsy to the matching adjusted model. Three models were evaluated in Columbia-GN, with the start time of kidney biopsy or clinical diagnosis being the (a) unadjusted model; (b) minimally adjusted model that included sex, pathologic diagnosis, age at biopsy, and *APOL1* kidney risk genotype; and (c) matching adjusted model that added hypertension, diabetes, use of RAAS inhibitor or immunosuppression at enrollment, and genetic ancestry cluster to the minimally adjusted model. Two models were evaluated in Columbia-CKD, with the start time as birth (a) unadjusted; and (b) matching adjusted model included *APOL1* kidney risk genotype, sex, hypertension, diabetes, use of RAAS inhibitor or immunosuppression at enrollment, and genetic ancestry cluster. Complete case sensitivity analysis was performed for CureGN models due to the use of imputed data. Complete case sensitivity analysis was also performed in the Columbia cohorts that included all individuals with available UACR (in mg/g) and eGFR data to evaluate for an impact of these clinical factors. Where UACR was missing, but UPCR or urine protein dip measurements were available, UACR was calculated using the crude estimation equations from Sumida et al. (56).

The maximally adjusted Cox’s proportional hazard model results from all 3 cohorts were meta-analyzed using random effects–restricted maximum likelihood (REML) to evaluate the effect of monogenic glomerular disorders on kidney failure risk and heterogeneity, including a subanalysis of only the glomerular disease cohorts.

Secondary analyses evaluated additional outcomes, including eGFR decline and probability of achieving disease remission, and sought to replicate the association of *APOL1* genotypes with adverse clinical outcomes. The effect of monogenic glomerular disorders and high-kidney-risk *APOL1* genotypes on eGFR decline rate in the CureGN cohort was evaluated using linear mixed-effects modeling, allowing a random slope and intercept for each individual. GFR was estimated using the CKD Epidemiology Collaboration 2021 formula without race if 25 years old or more or the Chronic Kidney Disease in Children Study U25_Cr_ formula if less than 25 years old (57, 58). Individuals with 2 creatinine measurements at least 90 days apart were included, yielding 7 measurements per individual (14,891 measurements, IQR of measurements per individual = 5–11). This model included the same covariates as the fully adjusted Cox’s proportional hazard model.

The effect of monogenic glomerular disorders and high-kidney-risk *APOL1* genotypes on the risk of not achieving CR (defined prospectively in CureGN as urine protein excretion by any method of <0.3 g/day) was evaluated within the CureGN cohort using logistic regression. This was adjusted for the same covariates as the eGFR decline analysis (43).

We evaluated the risk of kidney failure associated with specific high-risk *APOL1* genotypes using meta-analysis of the maximally adjusted Cox’s proportional hazard models of all 3 cohorts. We also evaluated the risk of kidney failure associated with high-risk *APOL1* genotypes in only those genetic ancestry clusters with individuals who carried a high-risk genotype.

We performed unadjusted and fully adjusted Cox’s proportional hazard modeling to evaluate the risk of kidney failure based on monogenic glomerular disorders and *APOL1* kidney risk genotype in phenotype-specific subgroups of the CureGN and Columbia-GN cohorts using the same models outlined above.

We evaluated the impact of ACMG SFs, using the structured data available in the CureGN cohort to identify individuals with a personal or family history of associated ACMG SF conditions based on the phenotype category of the SF gene’s effect using logistic regression. The risk of kidney failure was evaluated in those with ACMG SFs using the same covariates as the fully adjusted CureGN model. All positive cases were reviewed and the potential clinical impacts of these conditions on nephrology care were predicted.

All statistical analyses were performed in R version 4.3.1 (R Core Team).

### Study approval.

Within CureGN, each site obtained approval from an institutional review board, and informed consent and assent were obtained from participants and legal guardians. The CUICM study obtained institutional review board approval and informed consent was obtained from all included participants. Written consent was required from all participants. For patients unable to give consent themselves a parent or guardian provided consent in addition to participant assent.

### Data availability.

Data for CureGN are available to consortium members, and diagnostic variants identified from the CUIMC cohorts will be submitted to the NCBI ClinVar database. CureGN genetic data are available in dbGaP with accession number phs002480.v3.p3. Raw data for all graphs are reported in the Supporting Data Values file.

## Author contributions

MDE and AGG had full access to all the data in the study and take responsibility for the integrity of the data and the accuracy of the data analysis. MDE, AGG, MK, and KK conceptualized and designed the study. MDE, NV, MM, EC, SB, KB, NS, FZ, MV, CW, GJ, VK, MS, RGP, and SSC acquired, analyzed, or interpreted data. MDE and AGG wrote the original draft of the manuscript. MDE performed statistical analyses. PAC, ASB, SSC, LMGW, JH, BWG, BMR, JBK, MNR, WES, LAG, LBH, RJF, AP, LHM, MK, KK, and AGG provided consortium support. AGG supervised the study.

## Supplementary Material

Supplemental data

ICMJE disclosure forms

Supplemental table 1

Supplemental table 2

Supplemental table 3

Supporting data values

## Figures and Tables

**Table 1:**
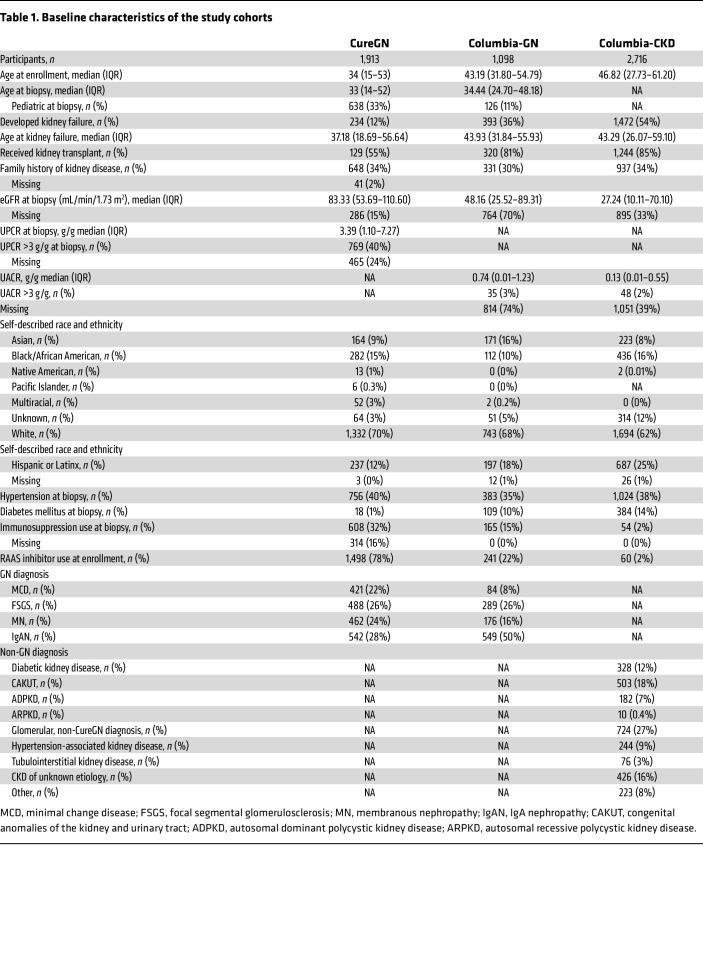
Baseline characteristics of the study cohorts

**Table 2:**
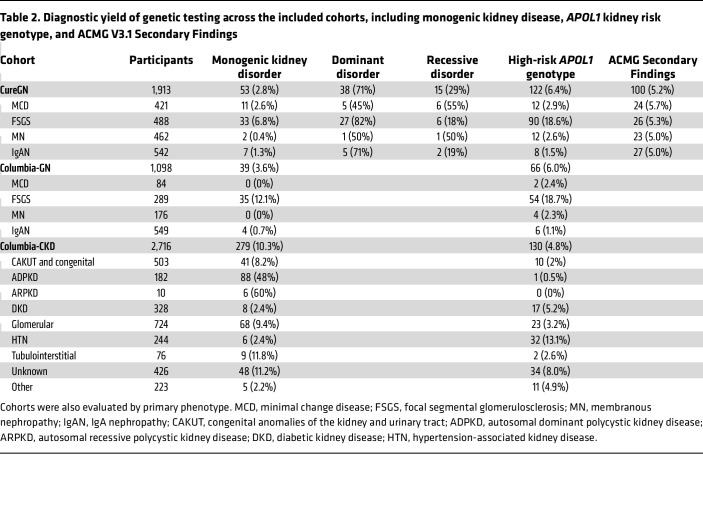
Diagnostic yield of genetic testing across the included cohorts, including monogenic kidney disease, *APOL1* kidney risk genotype, and ACMG V3.1 Secondary Findings

**Table 3:**
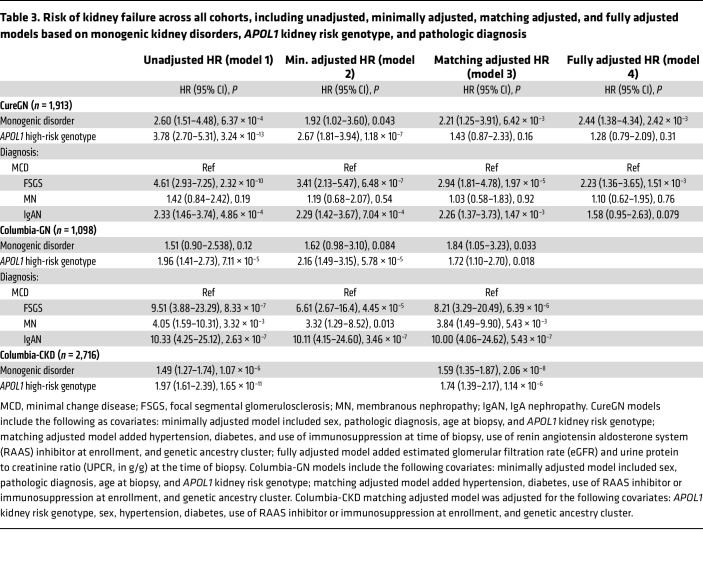
Risk of kidney failure across all cohorts, including unadjusted, minimally adjusted, matching adjusted, and fully adjusted models based on monogenic kidney disorders, *APOL1* kidney risk genotype, and pathologic diagnosis

## Version 1 09/03/2024

Electronic publication
