# Supplementary material for: Increased risk of kidney failure in patients with genetic kidney disorders
Source: J Clin Invest. 2024 Sep 3;134(17):e178573. doi: 10.1172/JCI178573 (PMC11364380; doi:10.1172/JCI178573)
Supplement: Supplemental data [file jci-134-178573-s018.pdf]

Supplementary Files:

Supplementary Figure 1. Conditionally adjusted event curves showing kidney failure rates based on monogenic kidney disorders in the Columbia-GN cohort presented at the same time scale as the CureGN cohort.

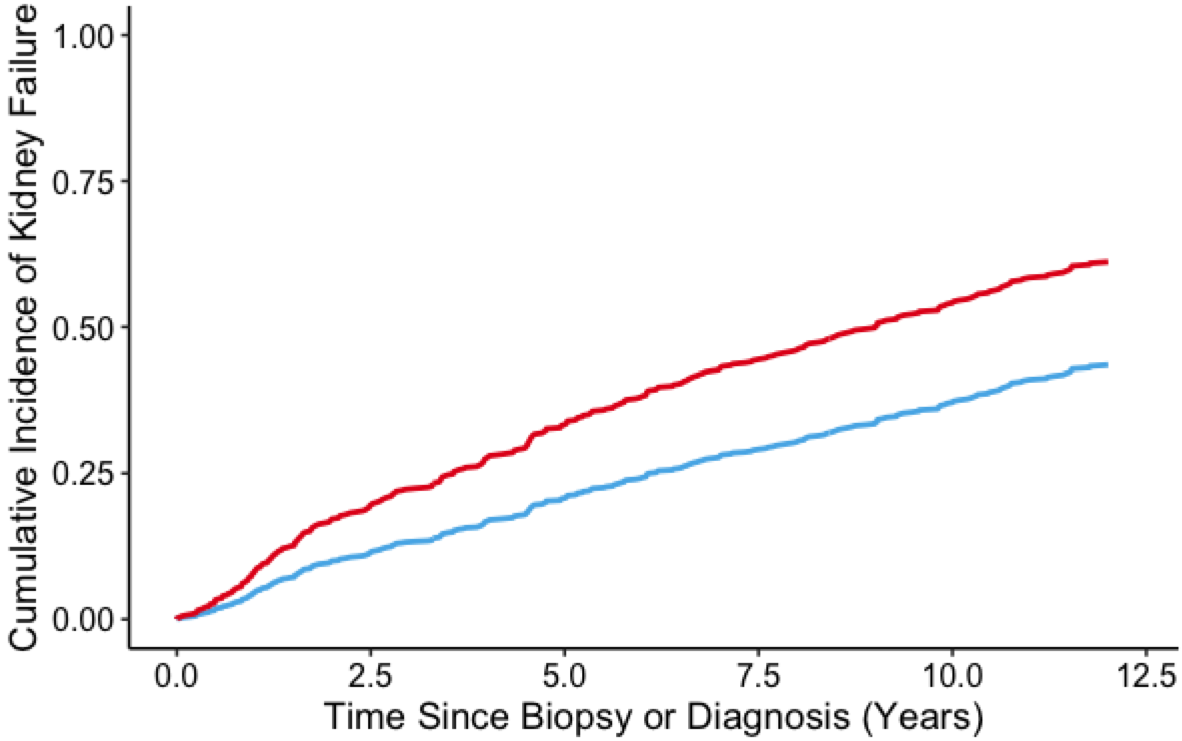

Supplementary Figure 2. Sensitivity analysis: restricted maximum likelihood random-effects meta-analysis of kidney failure risk across all three cohorts evaluating the effect of monogenic glomerular disorders using the fully adjusted Cox models and the use of the complete case analyses of Columbia-GN and Columbia-CKD that include eGFR and UACR values. Sub-analysis of genetic glomerular disorders within CureGN and Columbia-GN is also included.

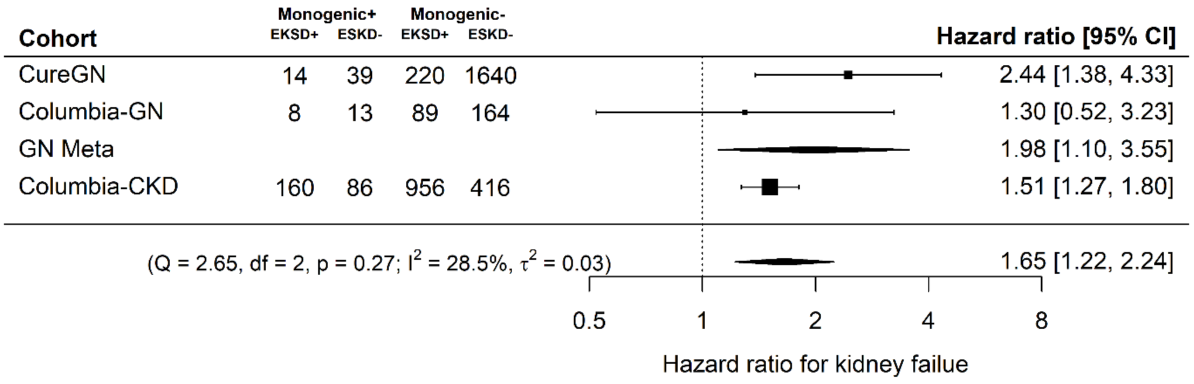

Supplementary Figure 3. Correlation plots between *APOL1* kidney risk genotype (x-axis) and genetic ancestry clusters (y-axis) using chi-squared tests across the 3 studies. Higher values show higher correlation strength.

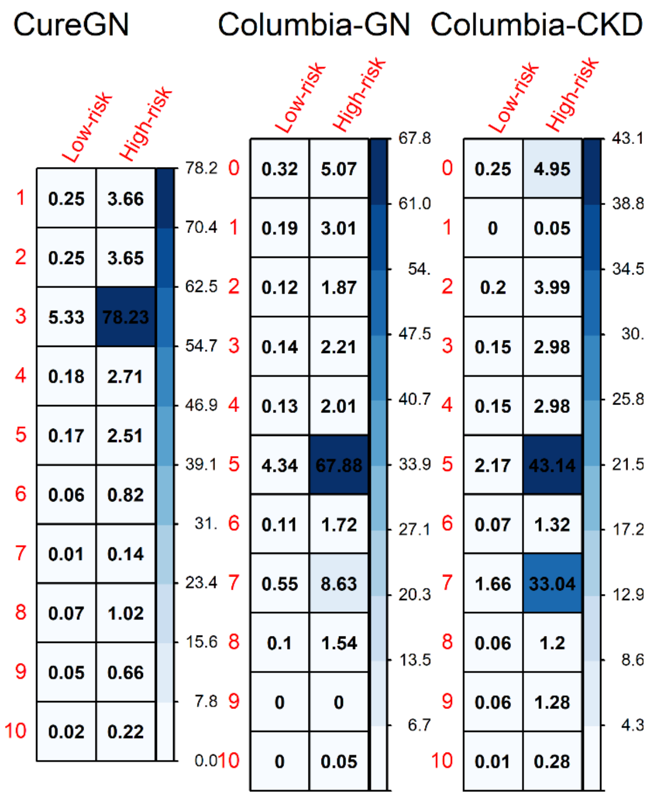

Supplementary Figure 4. Restricted maximum likelihood random-effects meta-analysis of kidney failure risk in the CureGN, Columbia-GN and Columbia-CKD cohorts based on specific *APOL1* kidney risk genotypes using the fully adjusted Cox models.

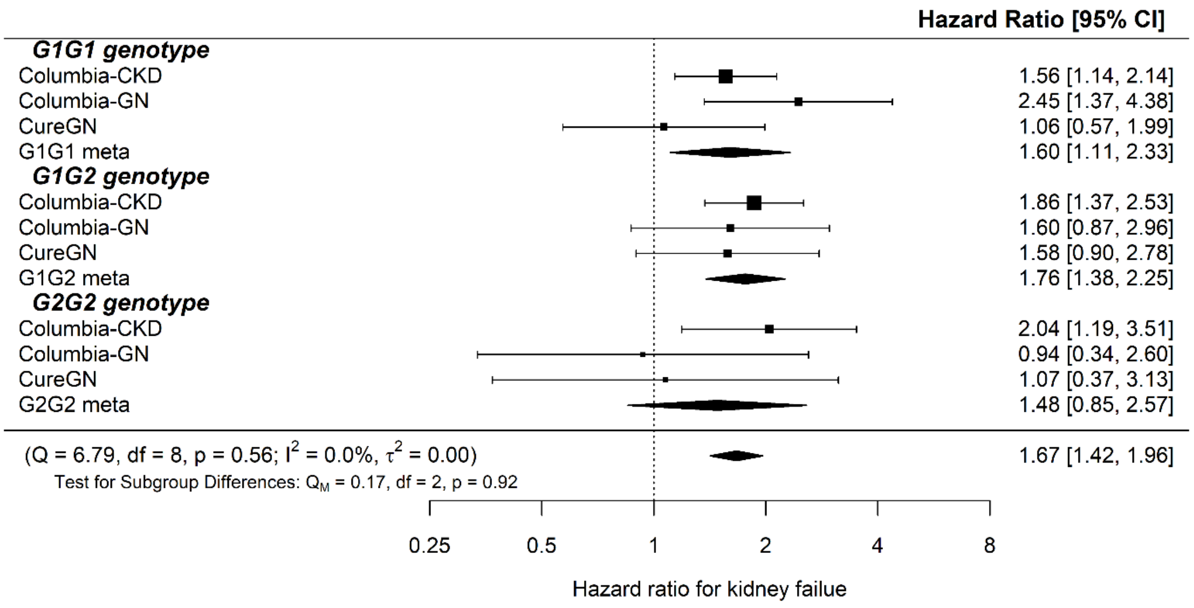

Supplementary Figure 5. Rate of eGFR decline in CureGN based on *APOL1* kidney risk genotype from time of kidney biopsy.

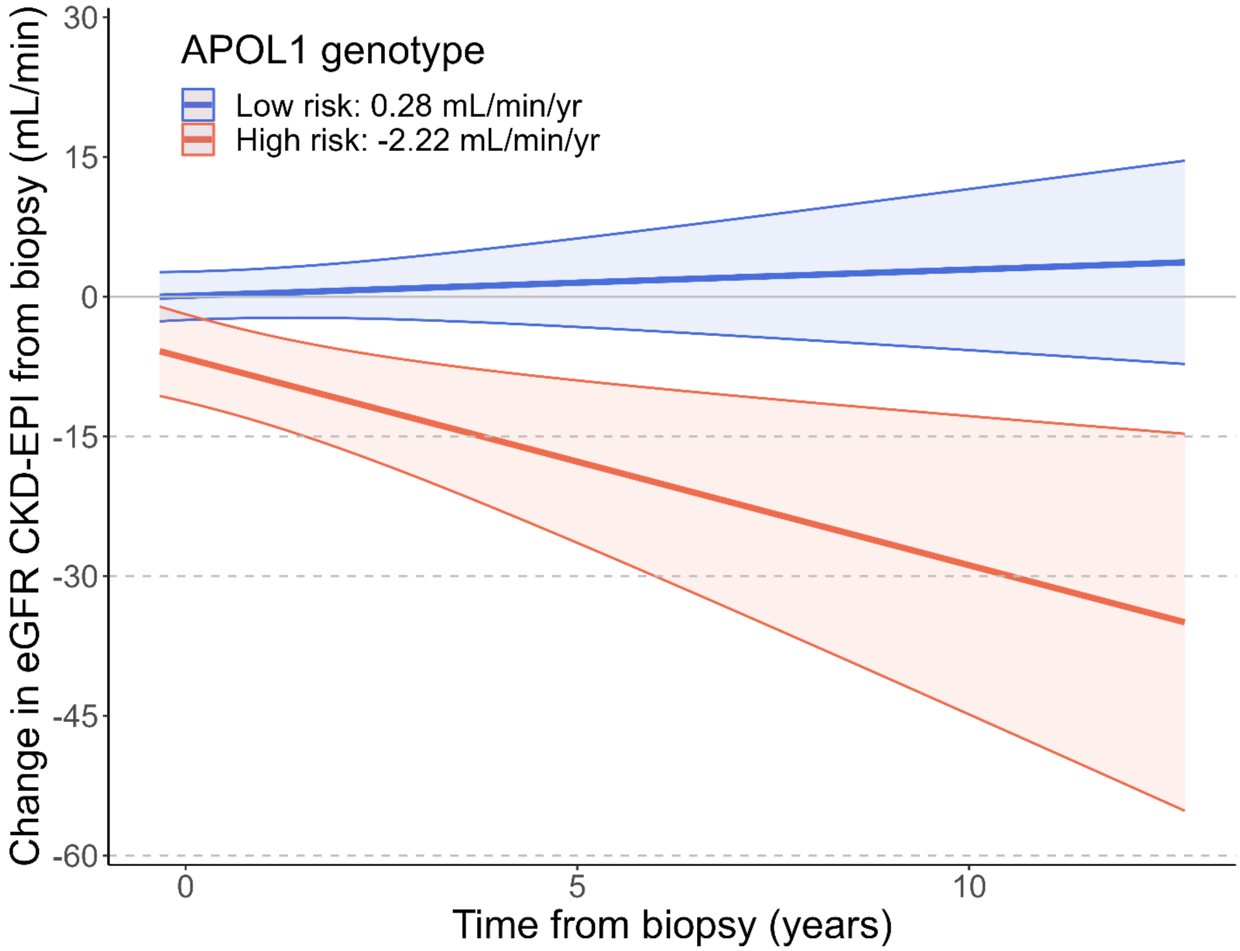

Supplementary Table 4: Expanded baseline characteristics by genetics features across the three cohorts. Abbreviations: minimal change disease (MCD), focal segmental glomerulosclerosis (FSGS), membranous nephropathy (MN), IgA nephropathy (IgAN), congenital anomalies of the kidney and urinary tract (CAKUT), autosomal dominant polycystic kidney disease (ADPKD), autosomal recessive polycystic kidney disease (ARPKD).

	CureGN					Columbia-GN				Columbia-CKD			
	Full Cohort	Monogenic	APOL1 High-risk	ACMG SF	No Genetic Disorder	Full Cohort	Monogenic	APOL1 High-risk	No Genetic Disorder		Monogenic	APOL1 High-risk	No Genetic Disorder
Participants, n	1913	53 (3%)	122 (6%)	100 (5%)	1651 (86%)	1098	39 (4%)	68 (6%)	997 (91%)	2716	279 (10%)	130 (5%)	2313 (85%)
Age at enrollment, median (IQR)	34 (15 - 53)	32 (14 - 46)	32 (17 - 46)	34 (17 - 57)	35 (15 - 54)	43.19 (31.80 - 54.79)	45 (29 - 54)	41 (30 - 47)	43 (32 - 55)	46.82 (27.73 - 61.20)	42 (28 - 58)	47 (31 - 57)	47 (27 - 62)
Age at biopsy, median (IQR)	33 (14 - 52)	30.8 (13.1 - 44.6)	29 (15.0 - 43.8)	33.3 (16.0 - 56.9)	33 (13.0 - 52.0)	34.44 (24.70 - 48.18)	37.7 (24.4 - 50.6)	35.84 (24.61 - 42.63)	33.85 (26.60 - 49.98)	NA	NA	NA	NA
Peditric at biopsy, n (%)	638 (33%)	17 (30%)	44 (36%)	27 (27%)	552 (33%)	126 (11%)	6 (15%)	3 (4%)	67 (7%)	NA	NA	NA	NA
Developed Kidney Failure, n (%)	234 (12%)	14 (26%)	41 (34%)	10 (10%)	176 (11%)	393 (36%)	15 (38%)	41 (60%)	343 (34%)	1472 (54%)	174 (62%)	108 (83%)	1195 (52%)
Age at kidney failure, median (IQR)	37.18 (18.69 - 56.64)	36.92 (19.80 - 49.88)	32.32 (19.50 - 48.07)	39.45 (21.32 - 61.22)	37.67 (18.45 - 57.49)	43.93 (31.84 - 55.93)	39.33 (29.70 - 56.83)	37.15 (28.07 - 43.44)	40.56 (31.10 - 51.17)	43.29 (26.07 - 59.10)	42.25 (28.20 - 55.84)	42.10 (29.36 - 51.63)	46.92 (31.21 - 58.77)
Received kidney transplant, n (%)	129 (55%)	10 (71%)	15 (37%)	4 (40%)	103 (59%)	320 (81%)	12 (80%)	36 (88%)	278 (81%)	1244 (85%)	151 (87%)	95 (88%)	1001 (84%)
Family history of kidney disease, n (%)	648 (34%)	32 (60%)	61 (50%)	28 (28%)	532 (32%)	331 (30%)	18 (46%)	22 (32%)	294 (29%)	937 (34%)	179 (64%)	51 (39%)	711 (31%)
Missing	41 (2%)	0 (0%)	0 (0%)	1 (1%)	40 (2%)		0 (0%)	0 (0%)	0 (0%)		0 (0%)	0 (0%)	0 (0%)
eGFR at biopsy, mL/mn/1.73m2 median (IQR)	83.33 (53.69 - 110.60)	79.55 (52.90 - 114.51)	62.81 (37.35 - 85.95)	91.57 (53.00 - 115.01)	85.17 (55.03 - 111.78)	48.16 (25.52 - 89.31)	37.25 (20.59 - 91.68)	37.13 (24.44 - 63.16)	52.49 (26.64 - 90.91)	27.24 (10.11 - 70.10)	29.86 (11.68 - 84.11)	11.86 (5.90 - 50.28)	29.15 (10.32 - 69.96)
Missing	286 (15%)	5 (9%)	17 (14%)	10 (10%)	256 (16%)	764 (70%)	13 (33%)	38 (58%)	713 (72%)	895 (33%)	21 (8%)	26 (20%)	849 (37%)
UPCR at biopsy, g/g median (IQR)	3.39 (1.10 - 7.27)	4.45 (2.07 - 8.32)	3.54 (1.63 - 7.74)	3.07 (1.09 - 5.83)	3.36 (1.04 - 7.29)	NA	NA	NA	NA	NA	NA	NA	NA
UPCR >3 g/g at biopsy, n (%)	769 (40%)	24 (45%)	51 (42%)	42 (42%)	658 (40%)	NA	NA	NA	NA	NA	NA	NA	NA
Missing	465 (24%)	12 (23%)	33 (27%)	21 (21%)	401 (24%)								
UACR, g/g median (IQR)	NA	NA	NA	NA	NA	0.74 (0.01 - 1.23)	1.23 (0.66 - 2.51)	1.18 (0.49 - 1.42)	0.55 (0.01 - 1.23)	0.13 (0.01 - 0.55)	0.07 (0.01 - 0.37)	0.34 (0.07 - 0.96)	0.14 (0.01 - 0.63)
UACR >3 g/g, n (%)	NA	NA	NA	NA	NA	35 (3%)	4 (10%)	3 (5%)	28 (3%)	48 (2%)	5 (2%)	3 (2%)	40 (2%)
Missing						814 (74%)	16 (41%)	46 (70%)	752 (76%)	1051 (39%)	20 (7%)	31 (24%)	1001 (43%)
Self-described race and ethnicity													
Asian, n (%)	164 (9%)	2 (4%)	0 (0%)	12 (12%)	150 (9%)	171 (16%)	4 (10%)	0 (0%)	167 (17%)	223 (8%)	17 (6%)	1 (1%)	205 (9%)
Black/African American, n (%)	282 (15%)	4 (8%)	106 (87%)	19 (19%)	163 (10%)	112 (10%)	4 (10%)	45 (66%)	66 (7%)	436 (16%)	29 (10%)	95 (93%)	317 (14%)
Native American, n (%)	13 (1%)	1 (2%)	1 (1%)	0 (0%)	11 (1%)	0 (0%)	0 (0%)	0 (0%)	0 (0%)	2 (0.01%)	0 (0%)	0 (0%)	5 (0.2%)
Pacific Islander, n (%)	6 (0.3%)	1 (2%)	3 (2%)	0 (0%)	3 (0.2%)	0 (0%)	0 (0%)	0 (0%)	0 (0%)	NA	NA	NA	NA
Multiracial, n (%)	52 (3%)	1 (2%)	5 (4%)	2 (2%)	44 (3%)	2 (0.2%)	0 (0%)	0 (0%)	0 (0%)	0 (0%)	0 (0%)	0 (0%)	0 (0%)
Unknown, n (%)	64 (3%)	6 (11%)	2 (2%)	2 (2%)	55 (3%)	51 (5%)	1 (3%)	4 (6%)	61 (6%)	314 (12%)	31 (11%)	9 (7%)	280 (12%)
White, n (%)	1332 (70%)	38 (72%)	5 (4%)	65 (65%)	1225 (74%)	743 (68%)	30 (77%)	19 (28%)	697 (70%)	1694 (62%)	202 (72%)	25 (19%)	1468 (63%)
Self-described race and ethnicity													
Hispanic or Latinx, n (%)	237 (12%)	12 (23%)	14 (11%)	12 (12%)	201 (12%)	197 (18%)	9 (23%)	24 (35%)	169 (17%)	687 (25%)	53 (19%)	35 (27%)	600 (26%)
Missing	3 (0%)	0 (0%)	1 (1%)	0 (0%)	2 (0.1%)	12 (1%)	1 (3%)	2 (3%)	10 (1%)	26 (1%)	5 (2%)	2 (2%)	21 (1%)
Hypertension at biopsy, n (%)	756 (40%)	25 (47%)	53 (44%)	49 (49%)	639 (39%)	383 (35%)	15 (38%)	34 (50%)	336 (34%)	1024 (38%)	111 (40%)	61 (47%)	855 (37%)
Diabetes Mellitus at biosy, n (%)	18 (1%)	0 (0%)	0 (0%)	1 (1%)	17 (1%)	109 (10%)	3 (8%)	13 (19%)	93 (9%)	384 (14%)	20 (7%)	26 (20%)	338 (15%)
Immunsuppression use at biopsy, n (%)	608 (32%)	9 (17%)	32 (26%)	34 (34%)	536 (32%)	165 (15%)	1 (3%)	1 (1%)	163 (16%)	54 (2%)	0 (0%)	2 (2%)	52 (2%)
Missing	314 (16%)	12 (23%)	19 (16%)	13 (13%)	272 (16%)	0 (0%)	0 (0%)	0 (0%)	0 (0%)	0 (0%)	0 (0%)	0 (0%)	0 (0%)
RAAS inhibitor use at enrollment, n (%)	1498 (78%)	44 (83%)	103 (84%)	77 (77%)	1286 (78%)	241 (22%)	0 (0%)	3 (4%)	238 (24%)	60 (2%)	1 (0.4%)	1 (1%)	58 (3%)
GN Diagnosis													
MCD, n (%)	421 (22%)	11 (21%)	12 (10%)	24 (24%)	375 (23%)	84 (8%)	0 (0%)	2 (3%)	82 (8%)	NA	NA	NA	NA
FSGS, n (%)	488 (26%)	33 (62%)	90 (74%)	26 (26%)	349 (21%)	289 (26%)	35 (90%)	56 (82%)	204 (20%)	NA	NA	NA	NA
MN, n (%)	462 (24%)	2 (4%)	12 (10%)	23 (23%)	426 (26%)	176 (16%)	0 (0%)	4 (6%)	172 (17%)	NA	NA	NA	NA
IgAN, n (%)	542 (28%)	7 (13%)	8 (7%)	27 (27%)	501 (30%)	549 (50%)	4 (10%)	6 (9%)	539 (54%)	NA	NA	NA	NA
Non-GN Diagnsis													
Diabetic kidney Disease, n (%)	NA	NA	NA	NA	NA	NA	NA	NA	NA	328 (12%)	8 (3%)	17 (13%)	303 (13%)
Congenital or Mendelian, n (%)	NA	NA	NA	NA	NA	NA	NA	NA	NA	693 (26%)	133 (48%)	11 (8%)	551 (24%)
Glomerular, non-CureGN diagnosis, n (%)	NA	NA	NA	NA	NA	NA	NA	NA	NA	724 (27%)	68 (24%)	23 (18%)	633 (27%)
Hypertension-associated kidney disease , n (%)	NA	NA	NA	NA	NA	NA	NA	NA	NA	244 (9%)	6 (2%)	32 (25%)	206 (9%)
Tubulointerstitial kidney disease, n (%)	NA	NA	NA	NA	NA	NA	NA	NA	NA	76 (3%)	9 (3%)	2 (2%)	65 (3%)
CKD of unknown etiology, n (%)	NA	NA	NA	NA	NA	NA	NA	NA	NA	422 (16%)	44 (16%)	34 (26%)	347 (15)
Other, n (%)	NA	NA	NA	NA	NA	NA	NA	NA	NA	229 (8%)	11 (4%)	11 (8%)	208 (9%)

Supplementary Table 5. Predictors of monogenic kidney disorders and high-risk *APOL1* genotypes across all three cohorts.

	Adjusted OR Monogenic HR (95% CI), P	Adjusted OR APOL1 HR (95% CI), P
**CureGN (n = 1913)**		
Family History	2.80 (1.57 - 4.98), 4.61×10^{-4}	1.51 (0.91 - 2.49), 0.11
Age at biopsy	1.00 (0.999 0 1.00), 0.79	1.00 (0.999 0 1.001), 0.86
Pathology Diagnosis		
MCD	Ref	Ref
FSGS	2.78 (1.35 - 5.74), 5.61×10^{-3}	8.86 (4.29 - 18.29), 4.19×10^{-9}
MN	0.18 (0.04 - 0.84), 0.029	0.88 (0.33 - 2.30), 0.79
IgAN	0.45 (0.17 - 1.18), 0.10	1.67 (0.59 - 4.73), 0.33
Race		
White	Ref	Ref
Asian	0.42 (0.10 - 1.80), 0.24	-
Black/African American	0.28 (0.10 - 0.81), 0.019	167 (64 - 432), 3.49×10^{-25}
Native American	1.46 (0.17 - 12.81), 0.73	11.9 (1.18 - 119), 0.036
Pacific Islander	4.80 (0.46 - 50.57), 0.19	364 (46 - 2892), 2.70×10^{-8}
Multiracial	0.51 (0.07 - 3.89), 0.51	28.5 (7.50 - 109), 9.62×10^{-7}
Unknown	2.37 (0.74 - 7.60), 0.15	3.37 (0.56 - 20.3), 0.18
Ethnicity		
Not Hispanic.Latinx	Ref	Ref
Hispanic/Latinx	1.10 (0.46 - 2.62), 0.83	2.46 (1.01 - 5.95), 0.047
**Columbia-GN (n = 1098)**		
Family History	1.69 (0.85 - 3.37), 0.14	0.91 (0.46 - 1.79), 0.78
Age at biopsy	1.00 (1.00 - 1.00), 0.84	1.00 (1.00 - 1.00), 0.98
Pathology Diagnosis		
MCD	Ref	Ref
FSGS	-	5.27 (1.15 - 24.23), 0.033
MN	-	0.56 (0.09 - 3.54), 0.54
IgAN	-	0.42 (0.07 - 2.34), 0.32
Race		
White	Ref	Ref
Asian	1.32 (0.41 - 4.25), 0.64	-
Black/African American	0.67 (0.21 - 2.10), 0.49	28.88 (13.31 - 62.66), $<2 \times 10^{-16}$
Multiracial	0 (0 - 0), 1.00	-
Unknown	0.34 (0.04 - 2.73), 0.31	1.36 (0.40 - 4.64), 0.62
Ethnicity		
Not Hispanic.Latinx	Ref	Ref
Hispanic/Latinx	1.31 (0.56 - 3.07), 0.53	6.03 (2.66 - 13.69), 1.70×10^{-5}
**Columbia-CKD (n = 2716)**		
Family History	3.59 (2.73 - 4.71), <2 x 10-16	1.28 (0.86 - 1.90), 0.23
Kidney Diagnosis		
Diabetic kidney disease	Ref	Ref
Congenital or Mendelian	6.97 (3.34 - 14.58), 2.43×10^{-7}	0.45 (0.20 - 1.01), 0.052
Glomerular	3.81 (1.80 - 8.09), 4.89×10^{-4}	0.80 (0.41 - 1.58), 0.53
Hypertension	1.02 (0.34 - 2.99), 0.98	2.14 (1.11 - 4.14), 0.024
Tubulointerstitial	3.91 (1.43 - 10.68),7.77×10^{-3}	0.72 (0.15 - 3.40), 0.68
CKD of unknown cause	3.64 (1.67 - 7.93), 1.17×10^{-3}	1.99 (1.04 - 3.80), 0.038
Other	1.52 (0.57 - 4.05), 0.40	1.21 (0.53 - 2.77), 0.66
Race		
White	Ref	Ref
Asian	0.69 (0.40 - 1.20), 0.19	0.32 (0.04 - 2.40), 0.27
Black/African American	0.75 (0.48 - 1.17)	16.92 (10.57 - 27.09), $<2 \times 10^{-16}$
Multiracial	-	-
Native American	-	-
Unknown	0.75 (0.46 - 1.24), 0.26	1.26 (0.55 - 2.86), 0.58
Ethnicity		
Not Hispanic.Latinx	Ref	Ref
Hispanic/Latinx	0.72 (0.50 - 1.04), 0.08	1.83 (1.13 - 2.96), 0.014

Supplementary Table 6. Matching adjusted and complete case including UACR and eGFR cox proportional hazard models of kidney failure risk in the Columbia cohorts.

Columbia-GN	HR for Kidney Failure Matching Adjusted (n = 1098)			HR for Kidney Failure Fully Adjusted, Complete Case (n=274)		
	HR	(95%CI)	P	HR	(95%CI)	P
Monogenic Glomerular Disorder	1.84	(1.05 to 3.23)	3.32E-01	1.30	(0.52 to 3.23)	5.75E-01
APOL1 high-risk genotype	1.72	(1.10 to 2.70)	1.80E-02	1.00	(0.41 to 2.46)	9.98E-01
Pathology Diagnosis:						
FSGS	8.21	(3.29 to 20.49)	6.39E-06	7.60	(0.96 to 59.93)	5.43E-01
MN	3.84	(1.49 to 9.90)	5.43E-03	2.55	(0.31 to 21.06)	3.85E-01
IgAN	10.00	(4.06 to 24.62)	5.43E-07	3.97	(0.50 to 31.78)	1.94E-01
Female Sex	0.98	(0.79 to 1.21)	8.30E-01	0.82	(0.52 to 1.28)	3.86E-01
Hypertension at biopsy	1.23	(0.99 to 1.52)	5.90E-02	1.55	(0.94 to 2.55)	8.91E-02
Diabetes at biopsy	0.90	(0.60 to 1.33)	5.90E-02	1.19	(0.55 to 2.58)	6.65E-01
RAAS inhibitor use at enrollment	0.75	(0.56 to 1.00)	5.20E-02	0.87	(0.48 to 1.59)	6.60E-01
Age at biopsy (Day)	1.00003	(1.00 to 1.00)	5.66E-04	0.9999	(0.9999 to 1.00)	1.24E-05
Immunosuppressive use at biopsy	3.33	(2.50 to 4.44)	<2E-16	0.87	(0.93 to 3.24)	8.15E-02
eGFR at biopsy	NA	NA	NA	0.95	(0.94 to 0.96)	<2E-16
UPCR at biopsy	NA	NA	NA	1	(0.9999 to 1.0002)	6.12E-01
Genetic Ancestry Cluster						
cluster1	1.34	(0.95 to 1.87)	9.28E-02	1.34	(0.63 to 2.85)	4.53E-01
cluster2	1.12	(0.75 to 1.68)	5.72E-01	1.11	(0.41 to 3.03)	8.41E-01
cluster3	1.28	(0.86 to 1.89)	2.17E-01	2.66	(1.07 to 6.64)	3.58E-02
cluster4	1.15	(0.76 to 1.75)	5.13E-01	0.69	(0.25 to 1.87)	4.67E-01
cluster5	1.89	(1.16 to 3.09)	1.06E-02	2.04	(0.70 to 5.97)	1.91E-01
cluster6	0.98	(0.65 to 1.48)	9.28E-01	1.67	(0.70 to 3.95)	2.46E-01
cluster7	1.41	(0.80 to 2.49)	2.38E-01	1.3	(0.38 to 4.42)	6.72E-01
cluster8	1.69	(1.10 to 2.61)	1.75E-02	4.15	(1.55 to 11.10)	4.55E-03
cluster9	2.06	(1.26 to 3.36)	4.00E-03	1.04	(0.37 to 2.96)	9.42E-01
cluster10	-	-	-	-	-	-

Columbia-CKD	HR for Kidney Failure Matching Adjusted (n = 2716)			HR for Kidney Failure Fully Adjusted, Complete Case (n=1618)		
	HR	(95%CI)	P	HR	(95%CI)	P
Monogenic Glomerular Disorder	1.59	(1.35 to 1.87)	2.06E-08	1.51	(1.27 to 1.80)	4.10E-06
APOL1 high-risk genotype	1.74	(1.39 to 2.18)	1.01E-06	1.48	(1.15 to 1.91)	2.51E-03
Female Sex	0.87	(0.79 to 0.97)	1.30E-02	1.07	(0.95 to 1.21)	2.65E-01
Hypertension at biopsy	0.88	(0.78 to 0.98)	2.10E-02	0.81	(0.71 to 0.93)	2.27E-03
Diabetes at biopsy	0.75	(0.65 to 0.87)	1.52E-04	0.85	(0.71 to 1.01)	7.14E-02
RAAS inhibitor use at enrollment	0.45	(0.28 to 0.74)	1.56E-03	0.32	(0.15 to 0.68)	2.84E-03
Immunosuppressive use	0.91	(0.56 to 1.49)	7.20E-01	1.45	(0.77 to 2.71)	2.49E-01
eGFR at biopsy	NA	NA	NA	0.99	(0.99 to 0.99)	<2E-16
UPCR at biopsy	NA	NA	NA	1.0001	(1.00 to 1.00)	1.55E-05
Genetic Ancestry Cluster						
cluster1	1.33	(1.12 to 1.57)	1.04E-03	1.22	(1.00 to 1.49)	4.58E-02
cluster2	0.6	(0.49 to 0.73)	6.14E-07	0.59	(0.46 to 0.75)	1.35E-05
cluster3	1.07	(0.87 to 1.32)	5.00E-01	0.93	(0.73 to 1.19)	5.54E-01
cluster4	0.87	(0.71 to 1.08)	2.07E-01	0.81	(0.63 to 1.04)	9.75E-02
cluster5	1.32	(1.07 to 1.62)	9.93E-03	1.01	(0.79 to 1.28)	9.52E-01
cluster6	1.12	(0.86 to 1.47)	3.93E-01	1.03	(0.77 to 1.39)	8.35E-01
cluster7	1.28	(1.01 to 1.62)	4.51E-02	1.05	(0.79 to 1.34)	7.56E-01
cluster8	1.24	(0.92 to 1.68)	1.61E-01	1.01	(0.70 to 1.46)	9.57E-01
cluster9	1.88	(1.41 to 2.51)	1.96E-05	1.69	(1.21 to 2.37)	2.25E-03
cluster10	0.97	(0.48 to 1.98)	9.40E-01	0.999	(0.49 to 2.04)	9.97E-01

Supplementary Table 7. Risk of kidney failure within specific glomerular subgroups of CureGN and Columbia-GN, including unadjusted, and fully adjusted Cox proportional hazard models based on monogenic glomerular disorders and *APOL1* kidney risk genotype.

	**Unadjusted HR**<br>**HR (95% CI), P**	**Full Adjusted HR**<br>**HR (95% CI), P**
**CureGN (n = 1913)**		
MCD (n = 421)		
Monogenic Disorder	3.54 (0.76 - 16.56), 0.10	4.47 (0.76 - 26.37), 0.08
APOL1 high-risk genotype	2.94 (0.63 - 13.79), 0.16	1.29 (0.12 - 13.91), 0.81
FSGS (n = 490)		
Monogenic Disorder	1.23 (0.59 - 2.56), 0.57	1.81 (0.83 - 3.94), 0.13
APOL1 high-risk genotype	2.12 (1.39 - 3.25), 6.72×10^{-4}	1.32 (0.69 - 2.55), 0.40
MN (n = 462)		
Monogenic Disorder	NA	NA
APOL1 high-risk genotype	11.20 (4.46 - 28.15), 7.79×10^{-6}	12.78 (2.51 - 65.18), $6.22 \times 10^{-}$[illegible]
IgAN (n = 542)		
Monogenic Disorder	5.96 (2.13 - 16.67), 9.36×10^{-4}	4.84 (1.27 - 18.56), 0.022
APOL1 high-risk genotype	2.25 (0.54 - 9.41), 0.26	0.51 (0.09 - 2.91), 0.44
**Columbia-GN (n = 1098)**		
MCD (n = 84)		
Monogenic Disorder	NA	NA
APOL1 high-risk genotype	5.98 (0.66 - 54.17), 0.11	NA
FSGS (n = 289)		
Monogenic Disorder	1.45 (0.84 - 2.49), 0.18	1.89 (1.02 - 3.49), 0.043
APOL1 high-risk genotype	1.57 (1.04 - 2.36), 0.032	1.38 (0.77 - 2.49), 0.28
MN (n = 176)		
Monogenic Disorder	NA	NA
APOL1 high-risk genotype	3.52 (0.83 - 14.82), 0.087	2.23 (0.26 - 19.27), 0.40
IgAN (n = 549)		
Monogenic Disorder	NA	NA
APOL1 high-risk genotype	8.35 (3.39 - 20.58), 4.01×10^{-6}	7.31 (2.53 - 21.15), 2.42×10^{-4}

Supplementary Table 8. Risk of not achieving complete remission within the CureGN cohort based on monogenic kidney disorder and *APOL1* kidney risk genotype. Adjusted for age at biopsy, sex, primary diagnosis, *APOL1* kidney risk genotype, hypertension, diabetes, eGFR, UPCR, use of immunosuppression at time of biopsy, genetic ancestry cluster, and use of RAAS inhibitor at enrollment.

	Unadjusted OR<br>OR (95%CI) P	Adjusted OR<br>OR (95%CI) P
Monogenic Glomerular Disorder	4.72 (2.49 - 8.92), 1.89×10^{-6}	5.25 (2.56 - 10.77), 6.31×10^{-6}
High-risk *APOL1* Genotypes	2.76 (1.85 - 4.11), 6.41×10^{-7}	1.36 (0.79 - 2.31), 0.27

Supplementary Table 9. Imputed dataset and complete case sensitivity analysis across the models within CureGN

CureGN	HR for Kidney Failure Minimally Adjusted, Imputed (n=1913)		HR for Kidney Failure Minimally Adjusted, Complete Case (n=1913)		HR for Kidney Failure Matching Adjusted, Imputed (n=1913)		HR for Kidney Failure Matching Adjusted, Complete Case (n=1599)		HR for Kidney Failure Fully Adjusted, Imputed (n=1913)		HR for Kidney Failure Fully Adjusted, Complete Case (n=1116)	
	HR (95%CI)	P	HR (95%CI)	P	HR (95%CI)	P	HR (95%CI)	P	HR (95%CI)	P	HR (95%CI)	P
Monogenic Glomerular Disorder	2.01 (1.15 to 3.51)	1.45E-02	2.01 (1.15 to 3.5)	1.38E-02	2.21 (1.25 to 3.91)	6.42E-03	2.36 (1.25 to 4.44)	8.01E-03	2.44 (1.38 to 4.34)	2.42E-03	3.36 (1.63 to 6.92)	1.01E-03
APOL1 high-risk genotype	2.72 (1.87 to 3.95)	2.97E-07	2.72 (1.88 to 3.94)	1.26E-07	1.43 (0.87 to 2.33)	1.58E-01	1.51 (0.9 to 2.53)	1.23E-01	1.28 (0.79 to 2.09)	3.12E-01	1.48 (0.76 to 2.86)	2.47E-01
Pathology Diagnosis:												
FSGS	3.40 (2.12 to 5.46)	6.73E-07	3.40 (2.13 to 5.44)	3.14E-07	2.94 (1.81 to 4.78)	1.97E-05	2.67 (1.61 to 4.40)	1.26E-04	2.23 (1.36 to 3.65)	1.51E-03	1.93 (1.03 to 3.64)	4.05E-02
MN	1.19 (0.68 to 2.08)	5.40E-01	1.19 (0.68 to 2.07)	5.39E-01	1.03 (0.58 to 1.83)	9.25E-01	1.03 (0.57 to 1.87)	9.25E-01	1.10 (0.62 to 1.95)	7.55E-01	1.01 (0.47 to 2.14)	9.87E-01
IgAN	2.29 (1.43 to 3.68)	6.85E-04	2.29 (1.43 to 3.67)	5.74E-04	2.26 (1.37 to 3.73)	1.47E-03	2.30 (1.37 to 3.86)	1.55E-03	1.58 (0.95 to 2.63)	7.91E-02	1.71 (0.89 to 3.27)	1.04E-01
Female Sex	0.75 (0.58 to 0.98)	3.48E-02	0.75 (0.58 to 0.98)	3.37E-02	0.73 (0.56 to 0.96)	2.35E-02	0.75 (0.56 to 1.00)	5.34E-02	0.73 (0.56 to 0.96)	2.41E-02	0.73 (0.51 to 1.05)	8.67E-02
Hypertension at biopsy					1.79 (1.34 to 2.4)	1.01E-04	1.64 (1.20 to 2.24)	1.93E-03	1.47 (1.10 to 1.96)	9.65E-03	1.57 (1.06 to 2.30)	2.30E-02
Diabetes at biopsy					0.39 (0.05 to 2.83)	3.49E-01	0.43 (0.06 to 3.08)	3.98E-01	0.33 (0.05 to 2.42)	2.74E-01	0.42 (0.06 to 3.10)	3.95E-01
RAAS inhibitor use at enrollment					1.67 (1.11 to 2.52)	1.51E-02	1.77 (1.04 to 3.01)	3.48E-02	1.77 (1.17 to 2.66)	6.85E-03	1.96 (1.04 to 3.67)	3.67E-02
Age at biopsy (Mo)	1.00 (1.00 to 1.00)	2.01E-02	1.00 (1.00 to 1.00)	1.92E-02	1.00 (1.00 to 1.00)	5.89E-01	1.00 (1.00 to 1.00)	5.63E-01	1.00 (1.00 to 1.00)	1.58E-03	1.00 (1.00 to 1.00)	3.24E-02
Immunosuppressive use at biopsy					1.07 (0.78 to 1.47)	6.69E-01	1.16 (0.84 to 1.6)	3.71E-01	1.09 (0.79 to 1.49)	6.03E-01	1.26 (0.84 to 1.89)	2.66E-01
eGFR at biopsy									0.98 (0.97 to 0.98)	6.41E-15	0.98 (0.97 to 0.99)	6.11E-10
UPCR at biopsy									1.03 (1.01 to 1.05)	4.26E-04	1.03 (1.01 to 1.06)	2.44E-03
Genetic Ancestry Cluster												
cluster2					1.13 (0.67 to 1.92)	6.41E-01	1.22 (0.69 to 2.17)	4.99E-01	1.30 (0.76 to 2.21)	3.40E-01	1.58 (0.78 to 3.18)	2.00E-01
cluster3					2.95 (1.7 to 5.11)	1.44E-04	3.30 (1.82 to 5.97)	7.87E-05	2.74 (1.58 to 4.77)	4.11E-04	3.47 (1.62 to 7.43)	1.36E-03
cluster4					1.71 (1.02 to 2.86)	4.26E-02	1.84 (1.03 to 3.26)	3.79E-02	1.83 (1.08 to 3.07)	2.37E-02	2.01 (0.97 to 4.18)	6.11E-02
cluster5					1.32 (0.76 to 2.3)	3.19E-01	1.38 (0.75 to 2.55)	3.02E-01	1.33 (0.76 to 2.34)	3.11E-01	1.44 (0.66 to 3.11)	3.60E-01
cluster6					1.83 (1.01 to 3.33)	4.75E-02	1.78 (0.91 to 3.46)	9.11E-02	1.94 (1.05 to 3.57)	3.32E-02	2.20 (0.97 to 4.96)	5.83E-02
cluster7					1.43 (0.74 to 2.77)	2.87E-01	1.68 (0.83 to 3.40)	1.45E-01	1.32 (0.68 to 2.58)	4.12E-01	1.29 (0.47 to 3.50)	6.21E-01
cluster8					1.60 (0.78 to 3.26)	1.97E-01	1.74 (0.81 to 3.71)	1.55E-01	2.03 (0.99 to 4.19)	5.38E-02	2.72 (1.15 to 6.42)	2.27E-02
cluster9					1.50 (0.65 to 3.49)	3.43E-01	1.81 (0.77 to 4.27)	1.76E-01	1.97 (0.83 to 4.64)	1.21E-01	1.92 (0.54 to 6.78)	3.12E-01
cluster10					2.97 (1.01 to 8.67)	4.69E-02	1.67 (0.39 to 7.22)	4.93E-01	3.08 (1.01 to 9.39)	4.77E-02	3.08 (0.65 to 14.66)	1.58E-01

Supplementary Table 10. Personal and family histories of ACMG V3.1 Secondary Findings associated conditions in CureGN subjects with ACMG secondary findings. Associations evaluated using logistic regression. American College of Medical Genetics (ACMG)

ACGM SF Category	Personal History of Complication n (%), P			Family History of Complication n (%), P		
Cancer	0/21	0%	0.99	7/21	33%	0.98
BRCA1/2 in females	0/5	0%	0.99	2/5	40%	0.75
Cardiovascular						
Aortopathy	0/6	0%	0.99	NA	NA	NA
Arrhythmia	3/18	17%	0.100	NA	NA	NA
Cardiomyopathy	0/23	0%	0.98	NA	NA	NA
Hypercholesterolemia	0/20	0%	0.98	9/20	45%	0.15
HNF1A and DM	0/5	0%	0.98	1/5	20%	0.60

Funding

Funding for the CureGN consortium is provided by U24DK100845 (formerly UM1DK100845), U01DK100846 (formerly UM1DK100846), U01DK100876 (formerly UM1DK100876), U01DK100866 (formerly UM1DK100866), and U01DK100867 (formerly UM1DK100867) from the National Institute of Diabetes and Digestive and Kidney Diseases (NIDDK). Patient recruitment is supported by NephCure Kidney International.

Dates of funding for first phase of CureGN was 9/16/2013-5/31/2019.

CureGN Collaborators

The CureGN Consortium members listed below, from within the four Participating Clinical Center networks and Data Coordinating Center, are acknowledged by the authors as Collaborators.
**CureGN Principal Investigators; *CureGN Site Principal Investigators; #CureGN Lead Coordinators.

CureGN Participating Clinical Centers (PCC) through Columbia University:

Columbia University, New York, NY, US: Wooin Ahn, Gerald Appel, Paul Appelbaum, Revekka Babayev, Andrew Bomback, Pietro Canetta, Brenda Chan, Vivette Denise D'Agati, Samitri Dogra, Hilda Fernandez, Ali Gharavi**, William Hines, Syed Ali Husain, Namrata Jain, Krzysztof Kiryluk, Fangming Lin, Maddalena Marasa#, Glen Markowitz, Hila Milo Rasouly, Sumit Mohan, Nicola Mongera, Jordan Nestor, Thomas Nickolas, Jai Radhakrishnan, Maya Rao, Simone Sanna-Cherchi, Shayan Shirazian, Michael Barry Stokes, Natalie Uy, Anthony Valeri, Natalie Vena
University of Warsaw, Warszawa, Poland: Bartosz Foroncewicz, Barbara Moszczuk, Krzysztof Mucha*, Agnieszka Perkowska-Ptasińska
IRCCS Giannina Gaslini, Genoa, Italy: Gian Marco Ghiggeri*, Francesca Lugani

CureGN Participating Clinical Centers (PCC) through the Pediatric Nephrology Research Consortium:

Arkana Laboratories, Little Rock, AR, USA: Josephine Ambruzs, Helen Liapis
Children's Hospital of Michigan, Detroit, MI, USA: Rossana Baracco, Amrish Jain*
Children's Hospital of New Orleans/ LSU Health, New Orleans, LA, USA: Isa Ashoor, Diego Aviles*
Children's Mercy Hospital, Kansas City, MO, USA: Tarak Srivastava*
Children's National Medical Center, Washington DC, USA: Sun-Young Ahn*
Cincinnati Children's Hospital Cincinnati, OH, USA: Prasad Devarajan, Elif Erkan*, Donna Claes, Hillarey Stone
Connecticut Children's Medical Center, Hartford, CT, USA: Sherene Mason*
Duke Children's Hospital Medical Center, Durham, NC, USA: Rasheed Gbadegesin*
East Carolina University Brody School of Medicine, Greenville, NC, USA: Liliana Gomez-Mendez*
Emory University, Atlanta, GA, USA: Larry Greenbaum**, Chia-shi Wang, Hong (Julie) Yin
Helen DeVos Children's Hospital, Grand Rapids, MI, USA: Yi Cai*, Goebel Jens, Julia Steinke
Levine Children's Hospital/Atrium Health, Charlotte, NC, USA: Donald Weaver*
Lurie Children's Hospital, Chicago IL, USA: Jerome Lane*
Mayo Clinic, Rochester, MN, USA: Carl Cramer*
Medical College of Wisconsin, Milwaukee, WI, USA: Cindy Pan, Neil Paloian, Rajasree Sreedharan*
Medical University of South Carolina, Charleston SC, USA: David Selewski, Katherine Twombley*
Nationwide Children's Hospital, Columbus, OH, USA: Corinna Bowers#, Mary Dreher# Mahmoud Kallash*, John Mahan, Samantha Sharpe#, William Smoyer**
Oregon Health and Science University, Portland, OR, USA: Amira Al-Uzri*, Sandra Iragorri
Riley Children's Hospital, Indianapolis, IN, USA: Myda Khalid*
Cardinal Glennon Children's Medical Center/ St. Louis University, St. Louis, MO, USA: Craig Belsha*
Texas Children's Hospital, Houston, TX, USA: Joseph Alge*, Michael Braun, AC Gomez, Scott Wenderfer*
Texas Tech Health Sciences Center, Amarillo, TX, USA: Tetyana Vasylyeva*
Children's of Alabama, University of Alabama, Birmingham, AL, USA: Daniel Feig*
University of Colorado Children's Hospital, Colorado, Aurora, CO, USA: Gabriel Cara Fuentes, Melisha Hannah*
University of Iowa Children's Hospital, Iowa City, IA, USA: Carla Nester*
University of Kentucky, Lexington, KY, USA: Aftab Chishti*
University of Louisville, Louisville, KY, USA: Jon Klein**
Holtz Medical Center, University of Miami, Miami, FL, USA: Chryso Katsoufis, Wacharee Seeherunvong*
University of Minnesota Children's Hospital, Minneapolis, MN, USA: Michelle Rheault*
University of New Mexico Health Sciences Center, Albuquerque, NM, USA: Craig Wong*
University of Oklahoma Health Sciences Center, Oklahoma City, OK, USA: Nisha Mathews*
University of Virginia, Charlottesville, VA, USA: John Barcia*, Agnes Swiatecka-Urban
University of Wisconsin, Madison, WI, USA: Sharon Bartosh*
Vanderbilt Children's Hospital, Nashville TN, USA: Tracy Hunley*
Washington University in St. Louis, St. Louis, MO, USA: Vikas Dharnidharka*, Joseph, Gaut

CureGN Participating Clinical Centers (PCC) through the University of North Carolina:
Hôpital Maisonneuve-Rosemont, Montreal, Canada: Louis-Philippe Laurin*, Virginie Royal
Medical University of South Carolina, Charleston, SC, USA: Anand Achanti, Milos Budisavljevic*, Sally Self
Northwestern University, Chicago, IL, USA: Cybele Ghossein, Yonatan Peleg, Shikha Wadhwani*
Ohio State University, Columbus, OH, USA: Salem Almaani, Isabelle Ayoub, Tibor Nadasdy, Samir, Parikh, Brad Rovin*
University of Chicago, Chicago, IL, USA: Anthony Chang
University of Alabama at Birmingham, Birmingham, AL, USA: Huma Fatima, Bruce Julian, Jan Novak, Matthew Renfrow, Dana Rizk*
University of North Carolina Kidney Center, Chapel Hill, NC, USA: Dhruti Chen, Vimal Derebail, Ronald Falk**, Keisha Gibson, Dorey Glenn, Susan Hogan, Koyal Jain, J. Charles Jennette, Amy Mottl*, Caroline Poulton#, Manish Kanti Saha
Vanderbilt University, Nashville, TN, USA: Agnes Fogo, Neil Sanghani*
Virginia Commonwealth University, Richmond, VA, USA: Jason Kidd*, Selvaraj Muthusamy

CureGN Participating Clinical Centers (PCC) through the University of Pennsylvania:
MetroHealth Medical Center/Case Western Reserve University, Cleveland, OH, USA: Jeffrey Schelling*
Cedars-Sinai Health System, Los Angeles, CA, USA: Jean Hou
Children's Hospital of LA, Los Angeles, CA, USA: Kevin Lemley*, Warren Mika, Pierre Russo
Children's Hospital of Philadelphia, Philadelphia, PA, USA: Michelle Denburg, Amy Kogon, Kevin Meyers*, Madhura Pradhan
Cleveland Clinic, Cleveland, OH, CA: Raed Bou Matar*, John O'Toole*, John Sedor*
Cohen Children's Medical Center, New Hyde Park, NY, USA: Christine Sethna*, Suzanne Vento #
Johns Hopkins University, Baltimore, MD, USA: Mohamed Atta, Serena Bagnasco, Alicia Neu, John Sperati*
Lundquist Institute at Harbor-UCLA Medical Center, Torrance, CA, USA: Sharon Adler*, Tiane Dai, Ram Dukkipati
Mayo Clinic, Rochester, MN, USA: Fernando Fervenza*, Sanjeev Sethi
Montefiore Medical Center, The Bronx, New York, NY, USA: Frederick Kaskel, Kaye Brathwaite, Kimberly Reidy*
New York University, New York, NY, USA: Joseph Weisstuch, Ming Wu, Olga Zhdanova
NIDDK, Bethesda, MD, USA: Jurgen Heymann, Jeffrey Kopp*, Meryl Waldman, Cheryl Winkler
Spokane Providence Medical Center, Spokane, WA, USA: Katherine Tuttle*
Stanford University, Palo Alto, CA, USA: Jill Krissberg, Richard Lafayette*, Kamal Fahmeedah, Elizabeth Talley
Sunnybrook Health Sciences Centre, Toronto, Canada: Michelle Hladunewich*
The Hospital for Sick Children, Toronto, Canada: Rulan Parekh*
University Health Network, Toronto, Canada: Carmen Avila-Casado, Daniel Cattran*, Reich Heather, Philip Boll
University of Miami, Miami, FL, USA: Yelena Drexler, Alessia Fornoni*
University of Michigan, Ann Arbor, MI, USA: Brooke Blazius*, Jeffrey Hodgin, Andrea Oliverio
University of Pennsylvania, Philadelphia, PA, USA: Jon Hogan, Lawrence Holzman**, Matthew Palmer, Gaia Coppock
University of Pittsburgh School of Medicine, Pittsburgh, PA, USA: Blaise Abromovitz*, Michael Mortiz*
University of Washington, Seattle, WA, USA: Charles Alpers, J. Ashley Jefferson*
UT Southwestern, Dallas, TX, USA: Elizabeth Brown, Kamal Sambandam*, Bethany Roehm

Data Coordinating Center (DCC):
Arbor Research Collaborative for Health, Ann Arbor, MI, USA: John Graff
Cedar Sinai Medical Center, Los Angeles, CA, USA: Cynthia Nast
Duke University, Durham, NC, USA: Laura Barisoni
Northwestern University, Chicago, IL, USA: Abigail Smith
University of Michigan, Ann Arbor, MI, USA: Brenda Gillespie**, Bruce Robinson**, Matthias Kretzler, Laura Mariani**

Steering Committee Chair: Lisa M. Guay-Woodford, Children's Hospital of Pennsylvania, Philadelphia, PA, USA
